# Chorioretinal Atrophy after Spontaneous Resolution of Myopic Foveoschisis

**DOI:** 10.1155/2014/825906

**Published:** 2014-12-24

**Authors:** Antonio García-Ben, José Manuel García-Campos, María José Morillo Sanchez, Laura Cristina Figueroa-Ortiz

**Affiliations:** ^1^Department of Ophthalmology, Santiago de Compostela University Medical School, C/Ramon Baltar, s/n, Santiago de Compostela, 15706 Coruna, Spain; ^2^Department of Ophthalmology, Malaga University Medical School, 29010 Malaga, Spain

## Abstract

Myopic foveoschisis is one of the major complications of pathologic myopia, and it was most recently identified by new imaging modalities. During the natural evolution of this complication, anatomical and visual improvement without surgical intervention is an unusual course, and most of these eyes remain stable or progressively worsen. The authors report a case of a highly myopic eye that developed patchy chorioretinal atrophy after spontaneous resolution of myopic foveoschisis, which to the best of our knowledge has not been reported previously in the medical literature.

## 1. Introduction

Myopic foveoschisis or myopic traction maculopathy is not an uncommon complication of pathologic myopia, with incidence rates ranging from 8% to 34% in highly myopic eyes with posterior staphyloma [1–5]. Optical coherence tomography (OCT) is an indispensable tool for diagnosis because the presence of myopic foveoschisis can only be suspected ophthalmoscopically and angiographically in some cases [1, 6, 7]. Using OCT, myopic foveoschisis is observed as the splitting of the inner retina from the outer retinal layers, with multiple columnar structures connecting the split retinal layers [1]. The pathogenesis of myopic foveoschisis has not yet been clearly established, but posterior vitreous traction, poor elasticity of the internal limiting membrane, inflexibility of the retinal vessels and epiretinal membrane in association with an elongation of the axial length, and stretching of the retina due to staphyloma may cause myopic foveoschisis [2, 8–12]. During the natural course of the disease, anatomical and visual improvement without surgical intervention is an extremely rare phenomenon, and most of these eyes remain stable or progressively worsen slowly over time [1, 9, 13–15].

We herein report a case of a highly myopic eye that developed patchy chorioretinal atrophy after spontaneous resolution of myopic foveoschisis.

## 2. Case Report

**Figure 1:**
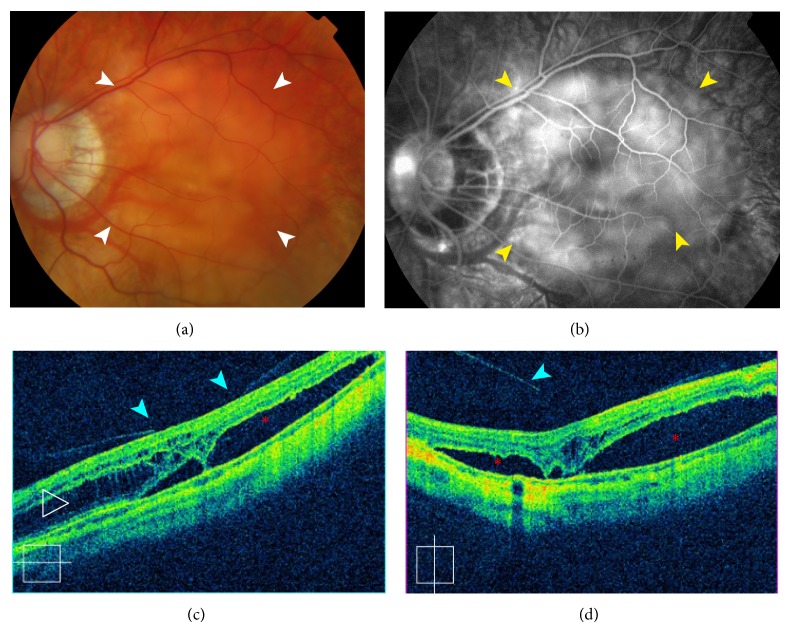
Color retinography, fluorescence angiography, and optical coherence tomography at the initial visit. (a) Left fundus photography showing a shallow retinal elevation between the temporal vascular arcades (white arrow) with peripapillary diffuse chorioretinal atrophy and a vertically elongated optic disc. (b) Late-phase fluorescein angiography image depicting irregular and diffuse hyperfluorescence at the posterior pole (yellow arrow). (c and d) Horizontal and vertical spectral-domain optical coherence tomography revealed the presence of myopic foveoschisis (open arrowheads) with a foveal retinal detachment (red asterisk) and a partially detached posterior hyaloid (blue arrow).

**Figure 2:**
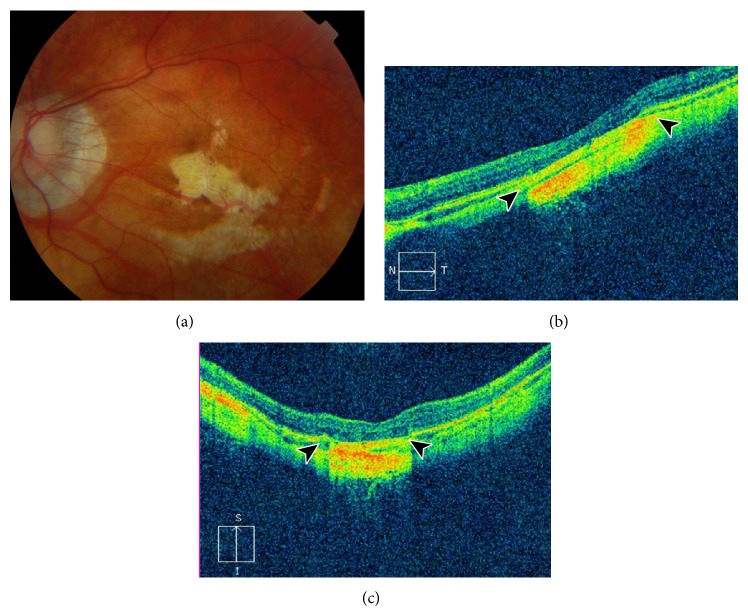
Color retinography and optical coherence tomography after twelve months. (a) Color photography illustrating a tessellated fundus with a yellowish, ill-defined, patchy chorioretinal atrophy in and around the macular area. (b and c) Horizontal and vertical spectral-domain optical coherence tomography showing complete resolution of the myopic foveoschisis and foveal retinal detachment with increased hyperreflectivity in the deep tissue of the sclera due to the atrophy of the superior retinal layers in the macular area (arrows). The outer retina, retinal pigment epithelium, and choroid are not present in the area of the patchy atrophy. The inner retina is directly attached to the sclera (between arrows).

A 20-year-old woman with a clinical diagnosis of pathologic myopia was referred to our hospital for blurred vision in her left eye. The mean spherical equivalents were −11 diopters in her right eye and −12 diopters in her left eye. The best-corrected visual acuity was 20/40 in the right eye and 20/200 in the left eye. She had no history of trauma, inflammation, infection, or surgery. No significant findings were observed in the anterior segment. Fundus biomicroscopy revealed an oval optic disc in both eyes and peripapillary diffuse atrophy, posterior staphyloma, and shallow retinal elevation in the left eye (Figure 1(a)). The OCT image of the left eye revealed the presence of a myopic foveoschisis with a foveal retinal detachment associated with a partial posterior vitreous detachment (Figures 1(c) and 1(d)). Fluorescein angiography showed irregular and nonspecific hyperfluorescence with dye leakage at the posterior pole and no evidence of choroidal neovascularization (Figure 1(b)). The option of vitrectomy was discussed; however, a conservative approach was chosen. The patient was followed up every 3 months. Twelve months later, she noticed a visual improvement in her left eye, although the best-corrected visual acuity remained unchanged at 20/200. Fundus and OCT examination showed patchy chorioretinal atrophy in and around the foveal area associated with complete resolution of the macular retinoschisis and foveal retinal detachment (Figure 2).

## 3. Discussion

Spontaneous resolution of myopic foveoschisis is an unusual event because most of these eyes remain stable or progress to more serious complications such as foveal retinal detachment or full-thickness macular hole formation [1, 9, 13–15]. The first description of spontaneous anatomical and visual improvement in highly myopic eyes with foveoschisis was reported by Polito et al. [12]. More recently, in a retrospective study by Shimada et al. [14], resolution was achieved in eight out of 207 eyes with myopic foveoschisis without any type of surgical intervention; specifically, a decrease was noted in two eyes, and complete resolution was achieved in six eyes. Six of the eight eyes with improvement in myopic foveoschisis showed release of retinal traction. Posterior vitreous detachment developed in four eyes, and spontaneous disruption of the detached internal limiting membrane developed in two eyes before resolution of myopic foveoschisis. In our case, OCT revealed the presence of a partial posterior vitreous detachment accompanied by myopic foveoschisis and a foveal retinal detachment. Twelve months later, the retina was completely reattached, and no vitreous structure was observed. Although the mechanism underlying the improvement in the anatomic and tomographic features of the macula is not well understood, it could be postulated that posterior vitreous detachment releases the anterior-posterior traction on the retina, leading to a resolution of the macular retinoschisis. This hypothesis is also supported by the clinical results of Hirota et al. [16], who demonstrated that the spontaneous release of the vitreofoveal anterior-posterior traction in three of four eyes with myopic foveoschisis led to reattachment of the inner to the outer retinal layer, removing the myopic schisis.

Most researchers believe that resolution of myopic traction maculopathy spontaneously or after vitrectomy with internal limiting membrane peeling or by placing an extramacular buckle causes anatomical and visual recovery [7, 12–14, 16, 17]. Although our patient noticed an improvement in her visual acuity, we did not observe any objective visual change after spontaneous resolution of the myopic traction maculopathy. The development of patchy chorioretinal atrophy in the macular area is likely to be responsible for this unexpected clinical course because myopic chorioretinal atrophy causes irreversible visual loss [17].

Myopic retinoschisis tends to develop in or around patchy atrophy, most likely due to a weak adhesion between the inner retina and sclera [2, 3, 18]. However, the chorioretinal atrophy in our case developed after regression of the myopic foveoschisis. The degenerative changes that are found in highly myopic eyes (e.g., posterior staphyloma and increased axial length) cause the obliteration of the choriocapillaris and a reduction of retinal blood flow, thus reducing the nutritional support for the retina, which subsequently atrophies [19, 20]. Although the exact mechanisms underlying the development of myopic chorioretinal atrophy after spontaneous resolution of myopic foveoschisis are unknown, the altered retinal blood flow in patients with myopic foveoschisis accompanied by the foveal retinal detachment demonstrated in previous studies could also play a role in the chorioretinal atrophy that occurred in our case [21]. However, a research study should be performed to confirm our hypothesis.
